# Risk factors and co-morbidities associated with changes in renal function among antiretroviral treatment-naïve adults in South Africa: A chart review

**DOI:** 10.4102/sajhivmed.v19i1.770

**Published:** 2018-04-12

**Authors:** Shirelle Assaram, Tivani P. Mashamba-Thompson, Nombulelo P. Magula

**Affiliations:** 1 Department of Internal Medicine, Nelson R. Mandela School of Medicine, University of KwaZulu-Natal, South Africa; 2 Department of Public Health Medicine, School of Nursing and Public Health, University of KwaZulu-Natal, South Africa

## Abstract

**Introduction:**

Our systematic scoping review has demonstrated a research gap in antiretroviral treatment (ART) nephrotoxicity as well as in the long-term outcomes of renal function for patients on ART in South Africa. Bearing in mind the high prevalence of human immunodeficiency virus (HIV) in South Africa, this is of great concern.

**Objectives:**

To determine the risk factors and co-morbidities associated with changes in renal function in HIV-infected adults in South Africa.

**Methods:**

We conducted a retrospective study of 350 ART-naïve adult patients attending the King Edward VIII HIV clinic, Durban, South Africa. Data were collected at baseline (pre-ART) and at six, 12, 18 and 24 months on ART. Renal function was assessed in the 24-month period using the Modification of Diet in Renal Disease equation and was categorised into normal renal function (estimated glomerular filtration rate [eGFR] ≥ 60), moderate renal impairment (eGFR 30–59), severe renal impairment (eGFR 15–29) and kidney failure (eGFR < 15 mL/min/1.73 m^2^). Generalised linear models for binary data were used to model the probability of renal impairment over the five time periods, controlling for repeated measures within participants over time. Risk ratios and 95% confidence intervals (CI) were reported for each time point versus baseline.

**Results:**

The cohort was 64% female, and 99% were Black. The median age was 36 years. At baseline, 10 patients had hypertension (HPT), six had diabetes, 61 were co-infected with tuberculosis (TB) and 157 patients had a high body mass index (BMI) with 25.4% being categorised as overweight and 19.4% as obese. The majority of the patients (59.3%) were normotensive. At baseline, the majority of the patients (90.4%) had normal renal function (95% CI: 86% – 93%), 7.0% (CI: 5% – 10%) had moderate renal impairment, 1.3% (CI: 0% – 3%) had severe renal impairment and 1.3% (CI: 0% – 3%) had renal failure. As BMI increased by one unit, the risk of renal impairment increased by 1.06 (CI: 1.03–1.10) times. The association of HPT with abnormal renal function was found to be insignificant, *p* > 0.05. The vast majority of patients were initiated on tenofovir disoproxil fumarate (TDF) (90.6%), in combination with lamivudine (3TC) (100%) and either efavirenz (EFV) (56.6%) or nevirapine (NVP) (43.4%).

**Conclusion:**

This study reports a low prevalence of baseline renal impairment in HIV-infected ART-naïve outpatients. An improvement in renal function after the commencement of ART has been demonstrated in this population. However, the long-term outcomes of patients with HIV-related renal disease are not known.

## Background

South Africa accounts for approximately 18% of global human immunodeficiency virus (HIV) infections with an estimated prevalence of 6.7 million HIV-infected people.^1^ There are almost 1000 new HIV infections, the majority of which are heterosexually transmitted.^2^ In an attempt to end the surge of HIV plaguing the African continent, the Joint United Nations Programme on HIV/AIDS (UNAIDS) has established an ambitious but achievable target to have 90% of all people tested for HIV and treated and virologically suppressed by 2020.^3^ Providing antiretroviral treatment (ART) to all people living with HIV (PLHIV) irrespective of CD4 count can help prevent HIV-related illness, avert acquired immune deficiency syndrome (AIDS)-related deaths and prevent new HIV infections.^3^ South Africa implemented the UNAIDS policy in September 2016.^4^ This universal access to ART for PLHIV is likely to lead to an increase in the burden of chronic diseases in South Africa as people are living longer with HIV.^5^

The 2014 study by Stanifer et al. has shown that chronic kidney disease (CKD) has been prevalent and is at an increase in sub-Saharan Africa, with 24% of HIV-infected patients having hypertension (HPT), 18.9% having diabetes and 10% having co-morbid CKD.^6^ The use of long-term medication for chronic illnesses can pose a threat to the kidneys, and the use of ART is no exception.^7,8,9^ Tenofovir disoproxil fumarate (TDF), a potentially nephrotoxic drug,^10,11^ is widely used as first-line ART in South Africa, and screening for baseline renal dysfunction prior to TDF initiation is essential.^12^ The South African ART guidelines recommend a serum creatinine and creatinine clearance at baseline (prior to ART initiation) and then at three months, six months and annually thereafter for patients on TDF.^13^

Our systematic scoping review has demonstrated a research gap in ART nephrotoxicity, particularly with TDF, as well as the long-term outcomes of renal function for patients on ART in South Africa. This is of great concern because we do not know the burden of ART nephrotoxicity or morbidity that our population may experience. Further, with the current health infrastructure, access to nephrologists and dedicated renal services are limited to a few tertiary hospitals. In this study, we aim to determine the risk factors and co-morbidities associated with changes in renal function in HIV-infected adults in South Africa. We anticipate that the results of this study will pave the way for prospective studies regarding the morbidity and mortality of ART nephrotoxicity and influence the expansion of renal services offered in the public health sector.

## Methodology

### Design

We conducted a retrospective study of 350 ART-naïve adult patients (18 years and older) attending the King Edward VIII HIV clinic, Durban, South Africa.

### Study population

Our study population was ART-naïve, HIV-infected adult patients who presented to us from across KwaZulu-Natal seeking initiation on ART. We included patients who were initiated on ART from April 2010 to December 2013 and followed up over a 24-month period. Patients already on ART who were transferred to the clinic were excluded.

### Data extraction

Clinical data were extracted from medical records of patients attending the HIV clinic at King Edward VIII Hospital in the study period from April 2010 to December 2013. Data were collected at baseline (pre-ART) and at six, 12, 18 and 24 months on ART. Data collected included socio-demographics, clinical parameters (weight, height, body mass index [BMI], blood pressure [BP], pulse, finger prick glucose and urine dipstick analysis), history of co-morbidities (HPT, diabetes, tuberculosis [TB] and pregnancy), laboratory data (serum creatinine and estimated glomerular filtration rate [eGFR]) and the type of ART regimen. We also documented patients who were lost to follow-up.

### Outcome measures

Chronic kidney disease was defined as either kidney damage or a GFR < 60 mL/min/1.73 m^2^ for ≥ three months irrespective of cause.^14^ Estimated GFR (eGFR) used in data analyses was calculated using the simplified Modification of Diet in Renal Disease (MDRD) equation without the ethnicity factor: eGFR (mL/min/1.73 m^2^) = 175 × [S_cr_(µmol/L)/88.4]^−1.154^ × Age (years)^−0.203^ × (0.742, if female)^15^ and was performed at the National Health Laboratory Services (NHLS) at King Edward VIII Hospital. The formula was adjusted to correct for the use of µmol/L as the unit of measure for serum creatinine.^15^ For this study, we modified the staging of CKD from that of the Kidney Disease Outcomes Quality Initiative (KDOQI) of the National Kidney Foundation (Table 1)^14^ because the NHLS reports creatinine clearance above 60 µmol/L as > 60 µmol/L and does not specify the actual eGFR level. For those patients who did not have a documented eGFR from the lab because either the age or gender was not documented on the lab request form, we manually calculated the value using the MDRD equation above, with age and gender captured during data collection. Proteinuria detected on urine dipstick analysis refers to the presence of protein in the urine.

**TABLE 1:** Staging of chronic kidney disease.

Description†	eGFR mL/min/1.73 m^2^
Normal renal function	≥ 60
Moderate renal impairment	30–59
Severe renal impairment	15–29
Kidney failure	< 15 (needs dialysis)

eGFR, estimated glomerular filtration rate.

† , An adaptation of the CKD classification of the KDOQI of National Kidney Foundation.

As per the South African Hypertension Guidelines, HPT is defined as a persistent elevation of BP ≥ 140/90 mmHg.^16^ The classification of HPT into four categories was made in accordance with the JNC8 guidelines (Table 2).^17^ Body mass index (BMI) is an index of weight-for-height that is used to classify persons as underweight, normal, overweight or obese. Body mass index is calculated as weight (kilograms)/height (metres)². Classification of BMI was based on that of the World Health Organization (WHO) Global Database on BMI (Table 3).^18^

**TABLE 2:** Classification of hypertension.

Classification†	Systolic blood pressure (mmHg)‡	-	Diastolic blood pressure (mmHg)
Normal	< 120	AND	< 80
Pre-hypertension	120–139	OR	80–89
Stage 1	140–159	OR	90–99
Stage 2	≥ 160	OR	≥ 100

*Source*: Hypertension: The Silent Killer: Updated JNC-8 Guideline Recommendations. Alabama Pharmacy Association, Montgomery, AL, USA; 2015.^17^

† , Classification as per JNC8 guidelines;

‡ , BP should be categorised according to the highest level of BP whether systolic or diastolic.

**TABLE 3:** Classification of body mass index.

Classification†	BMI‡ kg/m^2^
Underweight	< 18.50
Normal range	18.50–24.99
Overweight	≥ 25.00
Obese	≥ 30.00

BMI, body mass index.

† , Classification as per WHO guidelines;

‡ , BMI = weight (kilograms)/height (metres)^2^.

### Statistical analysis

IBM SPSS version 24 and Stata version 13 were used for data analysis. To assess the effect of time on ordinal outcomes, the outcomes were first categorised into binary variables indicating the presence or absence of a condition. eGFR categories were collapsed into normal (eGFR ≥ 60 mL/min/1.73 m^2^) versus renal impairment (moderate to kidney failure, eGFR < 60 mL/min/1.73 m^2^). Blood pressure categories were dichotomised to normal versus HPT – the latter included pre-hypertension. Generalised linear models for binary data were used to model the probability of renal impairment over the five time periods, controlling for repeated measures within participants over time. Risk ratios and 95% confidence intervals (CI) were reported for each time point versus baseline. A *p-*value of < 0.05 represented statistical significance.

#### Ethical consideration

Ethical approval was obtained from the KwaZulu-Natal Department of Health and from the University of KwaZulu-Natal (UKZN) Biomedical Research and Ethics Council (BREC) Ethics Committee. Full ethical approval was obtained from the BREC, and permission to conduct the research was granted by King Edward VIII Hospital management. All data generated or analysed during this study are included in this published article and its supplementary information files.

## Results

### Demographics

**FIGURE 1:**
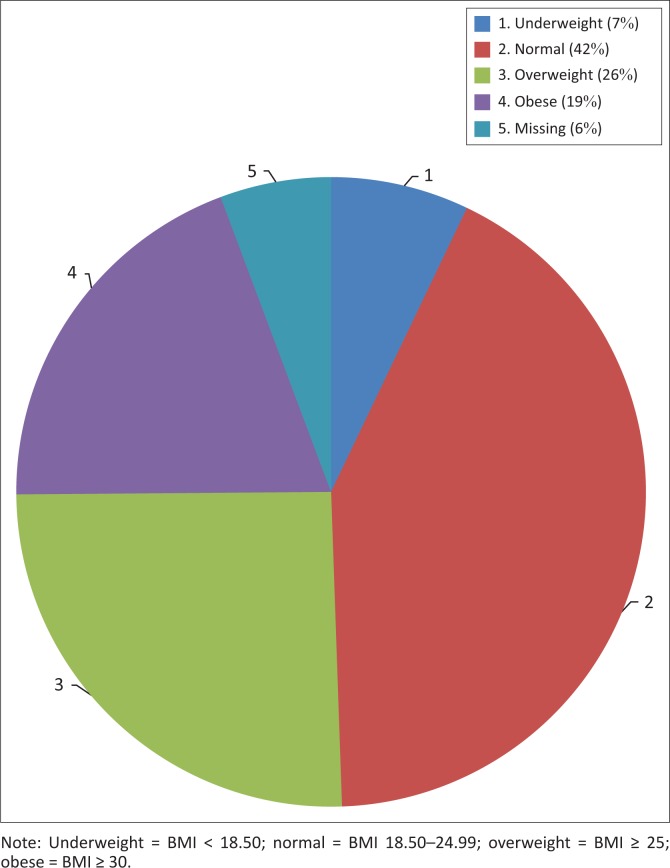
Body mass index categories of 350 patients at baseline.

**FIGURE 2:**
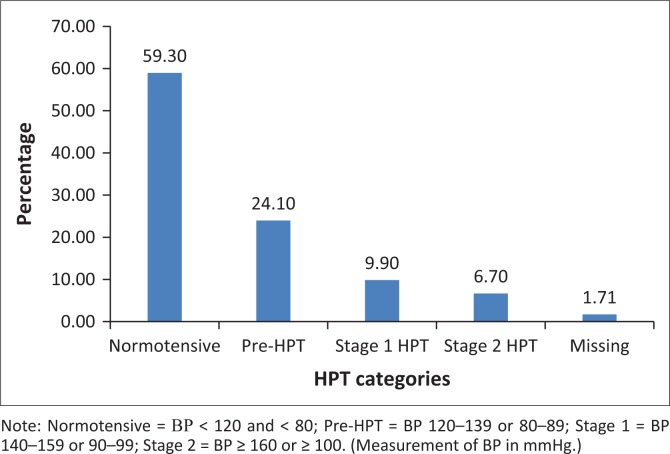
Hypertension categories of 350 patients at baseline.

The baseline characteristics of the 350 patients included in the analysis are illustrated in Table 4. Their mean age was 36.9 years, with a standard deviation of 9.7 years. The range was from 18 to 72 years, with a median age of 36 years. The cohort was 64% female, and 99% were Black. Of the women, 16% were pregnant at baseline. Also at baseline, 10 patients had HPT, six had diabetes, 14 had a combination of HPT and diabetes mellitus and 61 were co-infected with TB. At baseline, 157 patients had a high BMI, with 25.4% being categorised as overweight and 19.4% as obese (Figure 1). Because of a lack of documentation of either the weight or height or both, 20 patients did not have their BMI documented at baseline. The mean CD4 count at baseline was 177 cells/μL, with a standard deviation of 161.9 cells/μL. The majority of the patients (59.3%) were normotensive, while 24.1% were classified as pre-HPT, 9.9% as Stage 1 HPT and 6.7% as Stage 2 HPT (Figure 2).

**TABLE 4:** Baseline demographics of the 350 patients included in the analyses.

Variable	Category	*n*	%	95% CI
Gender	Female	224	64	0.59–0.69
	Male	126	36	0.31–0.41
Race	Black	348	99.4	0.98–1.00
	Indian	0	0	0.0
	White	0	0	0.0
	Mixed race	2	0.6	0.00–0.02
HPT	No	340	97.1	0.95–0.98
	Yes	10	2.9	0.02–0.05
Diabetes mellitus	No	344	98.3	0.96–0.99
	Yes	6	1.7	0.01–0.04
HPT and diabetes mellitus	Yes	14	0.6	0.001–0.02
TB	No	288	82.5	0.78–0.86
	Yes	61	17.5	0.14–0.22
Pregnant (women)	No	188	83.9	0.86–0.93
	Yes	36	16.1	0.07–0.14

CI, confidence intervals; HPT, hypertension; TB, tuberculosis.

### Baseline renal function

At baseline, the majority of the patients (90.4%) had a normal renal function (95% CI: 86% – 93%), 7.0% (CI: 5% – 10%) had moderate renal impairment, 1.3% (CI: 0% – 3%) had severe renal impairment and 1.3% (CI: 0% – 3%) had kidney failure (Table 5). The eGFR for 49 patients was not documented at baseline. Relative to baseline, the risk of developing renal failure decreased at the subsequent time points (Appendix 1). At six months, the risk of developing renal failure was 82% (CI: 5% – 72%) lower than baseline, at 12 months it was 56% (CI: 22% – 87%) lower, at 18 months it was 72% (CI: 10% – 77%) lower and at 24 months the risk was 49% (CI: 29% – 91%) lower than baseline.

**TABLE 5:** Categories of renal impairment from baseline to 24 months on antiretroviral treatment.

eGFR categories (eGFR mL/min/1.73 m^2^)	Baseline			6 months			12 months			18 months			24 months		
	*n*	%	CI	*n*	%	CI	*n*	%	CI	*n*	%	CI	*n*	%	CI
Normal (eGFR > 60)	272	90.4	0.86–0.93	112	98.2	0.93–1.00	181	95.8	0.92–0.98	143	97.3	0.93–0.99	212	95.1	0.91–0.97
Moderate RI (eGFR 30–59)	21	7.0	0.05–0.10	1	0.9	0.00–0.06	6	3.2	0.01–0.07	2	1.4	0.00–0.05	9	4.0	0.02–0.08
Severe RI (eGFR 15–29)	4	1.3	0.00–0.03	0	0.0	0	1	0.5	0.00–0.04	0	0.0	0	1	0.4	0.00–0.03
Kidney failure (eGFR < 15)	4	1.3	0.00–0.03	1	0.9	0.00–0.06	1	0.5	0.00–0.04	2	1.4	0.00–0.05	1	0.4	0.00–0.03

**Total**	**301**	**-**	**-**	**114**	**-**	**-**	**189**	**-**	**-**	**147**	**-**	**-**	**223**	**-**	**-**

eGFR, estimated glomerular filtration rate; CI, confidence interval, RI, renal impairment

Appendix 2 demonstrates the characteristics of the 29 patients with baseline renal impairment and depicts the trend of the eGFR over various time points. Of those patients with abnormal renal impairment at baseline, females predominated with renal impairment at 79%. Twenty-four per cent were co-infected with TB, while one patient had HPT. The average age of the 29 patients was 45 years. The median CD4 at baseline of these 29 patients was 147 cells/μL. Seven patients of the 29 patients (24%) with baseline renal impairment had persistent renal impairment at 24 months. Eight of the 29 patients did not have an eGFR done at 24 months. One patient was lost to follow-up at 24 months. The eGFR of 13 patients (44.8%) normalised at 24 months.

**FIGURE 3:**
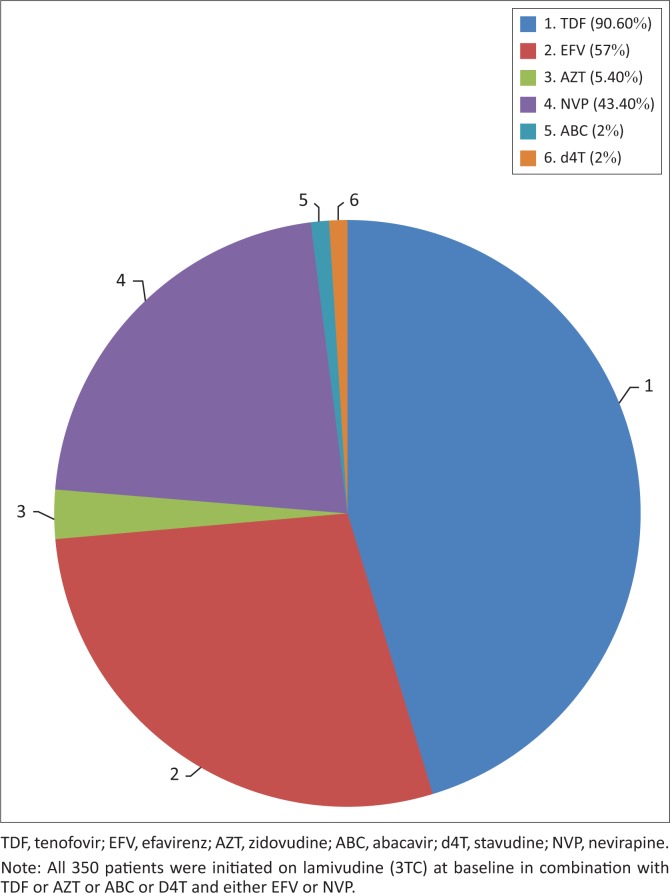
Distribution of antiretroviral treatment at treatment initiation.

In 2010, fixed-dose combinations were unavailable in the public sector; therefore, patients were initiated on individual antiretroviral agents. The vast majority of patients were initiated on TDF (90.6%), in combination with lamivudine (3TC) (100%) and either efavirenz (EFV) (56.6%) or nevirapine (NVP) (43.4%). Twenty-three of the 29 patients with baseline renal impairment (eGFR < 60 mL/min/1.73 m^2^) were commenced on a TDF-based regimen. Of the 10 patients with a baseline eGFR < 50 mL/min/1.73 m^2^ who were commenced on TDF, 7 had a normalisation of eGFR by 24 months, while three experienced deterioration in renal function. Two of the patients had TDF discontinued at 18 months and replaced with abacavir (ABC). The remaining patients with renal impairment were either initiated on ABC (three patients), zidovudine (AZT) (two patients) or stavudine (d4T) (one patient). Fourteen of the 36 pregnant women were initiated on AZT, while the remaining 22 were initiated on TDF. The remaining patients were initiated on either d4T, AZT or ABC by the attending clinician as first-line treatment with no reason specified. Figure 3 demonstrates this distribution of ART at baseline.

### Risk factors associated with abnormal renal function

The factors tested for association with abnormal renal function were age group, time, BMI and HPT. The factors which remained significant in the model are shown in Appendix 3. Compared with those aged under 30 years, there was no significant difference in renal impairment in those aged 30–49 years, *p* = 0.128 (CI: 2.51–1.52), while those aged ≥ 50 years were 6.8 (CI: 1.90–24.54) times more likely to develop renal impairment, *p* = 0.003. Time still remained a significant predictor. As time increased, the risk of abnormal renal function decreased. As BMI increased by one unit, the risk of renal impairment increased by 1.06 (CI: 1.03–1.10) times. A CD4 threshold of < 200 cells/μL had no significance to the association of impaired renal function, *p* = 0.378. The association of HPT with abnormal renal function was found to be insignificant over time, *p* = 0.194. Patients with HPT and aged ≥ 40 years in comparison with those with HPT and aged < 40 years did not show any significant risk for renal impairment, *p* = 0.226. Similarly, patients with HPT and a CD4 < 200 cells/μL did not have a significant risk for renal impairment compared with those patients with HPT and a CD4 ≥ 200 cells/μL, *p* = 0.384. A total of 21 patients (6%) were lost to follow-up over the two years, and 94% were retained in care.

### Urine dipstick analysis

Only five patients in the entire cohort had urine dipsticks performed at various time points (three at baseline, one at six months and one at 12 months). The test strip was positive for proteinuria in four of the five patients (80%), and three of these four patients (75%) with proteinuria were on a TDF-based regimen with a normal eGFR. The remaining patient had renal impairment and was on an ABC regimen.

## Discussion

We aimed to determine the risk factors and co-morbidities associated with changes in renal function in an adult cohort with HIV in South Africa. The cohort was mainly female and a large proportion of patients were either pregnant or co-infected with TB. The prevalence of renal impairment at baseline was found to be relatively low. Females predominated with baseline renal impairment and the majority of the patients were co-infected with TB. Of those patients with baseline renal impairment, the majority had a normalisation of their eGFR by 24 months. The risk of developing renal impairment decreased over the two-year period while patients were on ART. Risk factors found to be significant for renal impairment were older age, *p* = 0.003, and an increase in BMI, *p* < 0.001. In this cohort, HPT did not impact renal impairment significantly, *p* = 0.194, nor did a CD4 < 200 cells/μL, *p* = 0.378. Tenofovir disoproxil fumarate was the ART of choice for ART initiation along with 3TC and either NVP or EFV in keeping with South African National ART guidelines.^13^ Surprisingly, one-third of the patients with baseline renal impairment were initiated on a TDF-based regimen in contravention of the standard ART guidelines. These guidelines state that TDF should not be initiated in patients with an eGFR of < 50 mL/min/1.73 m^2^. This probably reflects the practice in clinics that may be understaffed where there may be a tendency for errors to occur.

Similar studies conducted in South Africa showed a low prevalence of baseline renal impairment in an HIV-infected cohort^12,19,20^ and some showed an improvement in eGFR after starting ART.^19,21,22^ Similar findings were noted in an African study by Stohr et al.^23^ carried out in Mozambique and Zimbabwe. As HIV has been shown to have a direct renal pathogenic role,^24,25^ we can deduce that treating the disease with ART diminishes the renal pathogenic effect. However, there is a lack of information on the long-term outcomes of renal disease in HIV-infected patients. Not all our patients experienced a resolution in their renal function as described earlier. Our cohort comprised mainly Black patients and therefore we were unable to compare the renal function of different ethnicities. Consensus regarding gender as a risk factor for renal dysfunction could not be arrived at, as some studies found women to be at greater risk,^19,26^ while others found men to be at greater risk.^20^ Older age has been documented in numerous studies including ours as a risk factor for renal impairment.^19,20,27^ A low CD4 count, high viral load and low haemoglobin are other variables found to be associated with a lower eGFR.^12,19,20,27^ In our study, despite having a mean CD4 of 177 cells/μL, a CD4 threshold of < 200 cells/μL was insignificant as a risk factor for renal impairment. Majority of the cohort had a baseline CD4 < 200 cells/μL in keeping with the National ART guidelines at the time (CD4 < 200 qualified for ART initiation).^13^ We did not assess a high viral load and low haemoglobin as variables in our study.

A high BMI has been documented in international literature as a modest risk factor for renal impairment over time.^27^ To the best of our knowledge, this is the first South African study to demonstrate BMI as a risk factor associated with changes in renal function in an HIV-infected cohort. This is a significant finding as a high BMI is also a risk factor for other chronic diseases such as HPT, diabetes and cardiovascular disease,^28,29^ which in turn are risk factors for CKD.^6^Although we did not find a significant link between HPT and renal impairment, this has been observed in other studies.^26,27^ A possible explanation for our findings could be the duration for which we collected data (two years) and ethnicity (our cohort mainly comprised Black patients), while in the other studies the duration was 96 weeks and 31 months, respectively, in predominantly White cohorts.^26,27^

Several studies have described TDF-associated nephrotoxicity as a pattern of renal injury involving the proximal renal tubule sometimes with Fanconi syndrome occurring together with decreased renal function.^7,30^ They recommend monitoring of renal function to prevent these outcomes.^10,30,31^ The South African ART guidelines recommend monitoring of renal function with urine dipsticks, a serum creatinine and eGFR levels at baseline, six months, 12 months and annually thereafter for patients on TDF.^13,32^ The rate of adherence to these guidelines is not known. In our study, we found missing serum creatinine and eGFR levels at six months, 12 months and 24 months. A possible explanation for this is that clinicians were attending to large clinic numbers with a poorly kept paper filing system, wherein these routine checks were missed. In addition, clinicians were missing the 12-month serum testing because they calculated the timing of this test as one year from the six-month blood test, which resulted in the test being taken at 18 months. Of those patients initiated on TDF with an eGFR < 50 mL/min/1.73 m^2^, only a few patients had deterioration in renal function, resulting in the discontinuation of TDF. Most likely this is because the majority had a reversible cause for the renal impairment, for example, dehydration rather than actual renal disease.

Urine analysis is important to detect proximal renal tubular damage in patients on TDF as well as undiagnosed HIV-associated nephropathy (HIVAN), yet they were rarely performed in our clinic setting, again, possibly because of large volumes of clinic patients combined with staff shortages and limited ablution facilities. Fabian et al.^33^ showed that urinary abnormalities were common in HIV-infected, ART-naïve outpatients and recommended that routine urinary screening of all new patients at ART clinics should be practiced. However, urine dipstick usage as a solitary screening tool is a topic with much debate, as research both locally and internationally suggests their poor validity in detecting proteinuria.^20,34^

A study conducted in South Africa by Han et al.^35^ showed that microalbuminuria alone could be an early marker for HIVAN. HIV-associated nephropathy is the most common histology seen on renal biopsy in South African patients.^21,22^ It is commonly found in Black patients who seem to have a genetic predisposition for the disease^36^ and Kasembeli et al.^37^ documented this finding in a South African cohort. Therefore, by deferring urine analysis, we may be missing an opportunity to detect early HIVAN in our predominantly Black high-risk cohort. The best method for urine screening is not known.^6^ Relying solely on serum creatinine levels and eGFR is inappropriate for this group of patients as HIVAN can occur with a normal renal function.^35^

It is important to highlight that when boosted with ritonavir or cobicistat, the renal toxicity profile of tenofovir tends to be worse.^30,38^ In this study, the majority of patients were treated with TDF, 3TC and either EFV or NVP, which tends to have a more favourable renal toxicity profile.^30,38^

A strength of this study we feel is that we had a good sample size of 350 and a good representation of the profile of patients who are currently attending ART clinics across South Africa. The main limitation of the study would be that of missing data, for example, the weight, height, BP and baseline serum creatinine were not documented in some patients. Lastly, we cannot comment on the diagnosis of those with persistent renal impairment at 24 months as renal biopsies were not done.

## Conclusion

This study shows a low prevalence of baseline renal impairment among HIV-infected, ART-naïve outpatients and an improvement in renal function after the commencement of ART. This highlights the possibility of HIV being the likely cause of the renal impairment at baseline. Caution should be exercised when excluding TDF as an option at initiation owing to renal impairment, as most patients have a reversible cause for the renal dysfunction which can be corrected before ART initiation. Ultimately, TDF as part of a fixed-dose combination is a once daily dose which greatly improves adherence. Long-term outcomes of patients with HIV-related renal disease are not known. Body mass index has been associated with the development of renal impairment in an HIV-infected cohort. With the recently introduced universal access to ART in South Africa, we anticipate the affliction of chronic diseases and their complications including renal disease to increase. Therefore, prospective studies targeting patients with HIV-related renal disease are needed in South Africa. Research on the most cost-effective and accurate urine diagnostics is also needed to detect early renal disease. Urine microalbumin screening is not routinely practiced at ART clinics despite being found to be an early marker for HIVAN.^35^ We need to improve and reinforce optimal primary care in our clinics, including regular BP monitoring, anthropometry and urine analysis. Lastly, we need to explore the influence of a high BMI in our HIV-infected patients and its link to CKD and other chronic diseases in order to promote a healthy lifestyle and dietician services as part of a holistic management of our patients (an area often neglected in clinical practice).

## Appendix 1

### Risk of developing renal impairment over time

Generalised linear models No. of obs = 973

Optimization : MQL Fisher scoring Residual df = 968

(IRLS EIM) Scale parameter = 1

Deviance = 401.4853798 (1/df) Deviance = 0.4147576

Pearson = 972.9991345 (1/df) Pearson = 1.005164

Variance function: V(u) = u*(1-u/1) [Binomial]

Link function : g(u) = ln(u) [Log]

BIC = -6258.726

(Std. Err. adjusted for 348 clusters in STUDYNO.)

------------------------------------------------------------------------------

 | Semirobust

 GFR_binary | Risk Ratio Std. Err. z P> |z| [95% Conf. Interval]

-------------+----------------------------------------------------------------

time |

6 months | 0.1820932 0.1277103 -2.43 0.015 0.0460582 0.7199133

12 months | 0.4393359 0.1536724 -2.35 0.019 0.2213403 0.8720329

18 months | 0.2824302 0.1451236 -2.46 0.014 0.1031646 0.7731993

24 months | 0.5142902 0.1508526 -2.27 0.023 0.2894223 0.9138699

|

_cons | 0.0963455 0.0170317 -13.24 0.000 0.0681331 0.1362401

## Appendix 2

**TABLE 1-A2:** Follow-up of estimated glomerular filtration rate of the 29 patients with baseline renal impairment.

Patient	Variable	Baseline	6 months	12 months	18 months	24 months
49/11 72 years	**eGFR** **CD4**	54.79 159	Nd Nd	> 60 75.04 178	Nd Nd	Nd 182
Female BMI 27.1	**ART**	TDF,3TC, EFV				
205/11 53 years	**eGFR** **CD4**	56.81 147	> 60 78.51 213	> 60 69.05 259	> 60 60.68 312	> 60 60.68 356
Female BMI 28.7	**ART**	TDF,3TC, EFV				
568/10 51 years	**eGFR** **CD4**	49.00 2.00	Nd Nd	Nd Nd	Nd Nd	> 60 86.41 233
Female BMI nd	**ART**	TDF,3TC, EFV				
275/11 32 years	**eGFR** **CD4**	50.46 109	Nd Nd	Nd Nd	> 60 124.02 345	Nd Nd
Female BMI 28.1	**ART**	TDF,3TC, EFV				
371/11 36 years	**eGFR** **CD4**	59.42 17	Nd Nd	> 60 105.45 41	> 60 108.88 31	Nd Nd
Male, TB BMI nd	**ART**	TDF,3TC, EFV				
567/11 59 years	**eGFR** **CD4**	46.13 143	Nd Nd	Nd Nd	> 60 63.40 146	40.61 161
Male BMI 21.3	**ART**	TDF,3TC, EFV				
578/11 51 years	**eGFR** **CD4**	58.00 147	Nd Nd	47.40 416	Nd Nd	44.95 539
Female, TB 34.60	**ART**	TDF,3TC, EFV				
587/11 56 years	**eGFR** **CD4**	38.45 227	Nd Nd	> 60 78.97 363	Nd Nd	> 60 72.68 436
Female BMI 46.5	**ART**	TDF,3TC, EFV				
788/11 40 years	**eGFR** **CD4**	51.65 236	Nd Nd	Nd Nd	> 60 154.48 50	Nd Nd
Male BMI 27	**ART**	TDF,3TC, EFV				
1033/11 60 years	**eGFR** **CD4**	47.95 324	Nd Nd	> 60 90.18 692	Nd Nd	Nd Nd
Female BMI 37.8	**ART**	TDF,3TC, EFV				
1111/11 36 years Female	**eGFR** **CD4**	52.59 277	> 60 86.38 634	> 60 82.12 424	> 60 70.40 413	> 60 68.42 447
BMI 26.4	**ART**	TDF,3TC, NVP	TDF,3TC,EFV			
1145/11 50 years	**eGFR** **CD4**	57.81 365	Nd Nd	> 60 63.45 438	> 60 62.11 518	> 60 72.79 516
Male BMI 20.7	**ART**	TDF,3TC, EFV				
819/10 33 years	**eGFR** **CD4**	3.68 13	4.01 Nd	Nd Nd	LTFU LTFU	LTFU LTFU
Male, HPT BMI 22.5	**ART**	ABC,3TC, EFV				
51/12 46 years	**eGFR** **CD4**	20.63 238	59.23 431	11.88 362	34.26 187	34.83 370
Female BMI 46.1	**ART**	TDF,3TC, EFV			ABC,3TC,EFV	
150/12 42 years	**eGFR** **CD4**	53.98 16	Nd Nd	> 60 73.50 49	> 60 79.59 78	Nd Nd
Female BMI 27.0	**ART**	TDF,3TC, NVP			AZT,3TC,Aluvia	
341/12 51 years	**eGFR** **CD4**	14.17 46	Nd Nd	Nd Nd	LTFU LTFU	15.78 227
Male BMI 32.3	**ART**	AZT,3TC, EFV	ABC,3TC,EFV			
346/12 49 years	**eGFR** **CD4**	25.33 128	Nd Nd	54.25 287	Nd Nd	43,82 331
Male, TB BMI 25.1	**ART**	ABC,3TC, EFV				
416/12 46 years	**eGFR** **CD4**	51.18 150	Nd Nd	> 60 71.06 418	Nd Nd	> 60 80.80 393
Female BMI 45.6	**ART**	TDF,3TC, EFV				FDC
475/12 30 years	**eGFR** **CD4**	37.98 20	Nd Nd	> 60 83.84 330	Nd Nd	> 60 117.48 410
Female, TB BMI 14.6	**ART**	TDF,3TC, EFV			FDC	
843/12 42 years	**eGFR** **CD4**	58.80 284	Nd Nd	> 60 61.12 475	Nd Nd	58.07 591
Female BMI 38.1	**ART**	TDF,3TC, EFV		FDC		
431/12 69 years	**eGFR** **CD4**	19.82 139	> 60 77.02 328	Nd Nd	> 60 71.96 519	> 60 73.17 700
Female, TB BMI 52.8	**ART**	ABC,3TC, EFV				
476/12 30 years	**eGFR** **CD4**	14.49 45	Nd Nd	> 60 83.84 330	Nd Nd	> 60 101.81 410
Female BMI 14.6	**ART**	TDF,3TC, EFV				
600/12 44 years	**eGFR** **CD4**	49.38 299	Nd 299	> 60 106.38 527	Nd Nd	> 60 85.89 637
Female BMI 42.5	**ART**	TDF,3TC, EFV				FDC
1039/10 40 years	**eGFR** **CD4**	39.70 193	Nd Nd	Nd Nd	> 60 69.91 600	> 60 76.59 566
Female BMI 24.8	**ART**	AZT,3TC, NVP				
1266/10 49 years	**eGFR** **CD4**	24.22 218	Nd 287	24.82 367	Nd Nd	Nd Nd
Female, TB BMI 37.4	**ART**	TDF,3TC, EFV			ABC,3TC,EFV	
1273/10 47 years	**eGFR** **CD4**	51.55 190	LTFU LTFU	LTFU LFTU	LTFU LFTU	Nd 191
Female BMI 47.5	**ART**	TDF,3TC, EFV				
494/10 26 years	**eGFR** **CD4**	50.48 36	Nd Nd	> 60 100.92 246	Nd Nd	> 60 77.43 198
Female BMI 22.8	**ART**	D4T,3TC, EFV		D4T,3TC,NVP		
1336/10 49 years	**eGFR** **CD4**	56.99 163	Nd Nd	> 60 99.80 222	Nd Nd	6.90 298
Female BMI 21.3	**ART**	TDF,3TC, EFV				
1339/10 25 years	**eGFR** **CD4**	46.24 71	> 60 110.06 Nd	Nd Nd	Nd Nd	> 60 125.36 299
Male, TB BMI 19.4	**ART**	TDF,3TC, EFV				

Nd, not done; ART, antiretroviral treatment; TB, tuberculosis; BMI, body mass index; LTFU, lost to follow-up; FDC, fixed-dose combination.

## Appendix 3

### Risk factors and co-morbidities associated with abnormal renal function

Generalised linear models No. of obs = 972

Optimization : MQL Fisher scoring Residual df = 963

 (IRLS EIM) Scale parameter = 1

Deviance = 353.6712349 (1/df) Deviance = 0.3672598

Pearson = 991.4235583 (1/df) Pearson = 1.029516

Variance function: V(u) = u*(1-u/1) [Binomial]

Link function : g(u) = ln(u) [Log]

 BIC = -6271.148

 (Std. Err. adjusted for 348 clusters in STUDYNO)

------------------------------------------------------------------------------

| Semirobust

GFR_binary | Risk Ratio Std. Err. z P> |z| [95% Conf. Interval]

-------------+----------------------------------------------------------------

time |

6 months | 0.1905768 0.133623 -2.36 0.018 0.0482225 0.7531656

12 months | 0.4020471 0.1349437 -2.71 0.007 0.2082468 0.7762033

18 months | 0.2356236 0.1224814 -2.78 0.005 0.0850646 0.6526627

24 months | 0.4218433 0.1191978 -3.05 0.002 0.2424556 0.7339563

|

BMI | 1.060231 0.0130867 4.74 0.000 1.03489 1.086194

|

HPT |

yes | 1.891108 0.9282237 1.30 0.194 0.7226302 4.94899

|

age group |

2 | 2.512881 1.519828 1.52 0.128 0.767985 8.22226

3 | 6.822283 4.45519 2.94 0.003 1.896991 24.53546

|

_cons | 0.0066735 0.0045581 -7.33 0.000 0.0017497 0.0254532

------------------------------------------------------------------------------

### HPT categories as risk factors for abnormal renal function

Generalised linear models No. of obs = 38

Optimization : MQL Fisher scoring Residual df = 32

(IRLS EIM) Scale parameter = 1

Deviance = 213.9306761 (1/df) Deviance = 6.685334

Pearson = 500000047.4 (1/df) Pearson = 1.56e+07

Variance function: V(u) = u*(1-u/1) [Binomial]

Link function : g(u) = ln(u) [Log]

 BIC = 97.52792

 (Std. Err. adjusted for 20 clusters in STUDYNO)

------------------------------------------------------------------------------

| Semirobust

GFR_binary | Risk Ratio Std. Err. z P> |z| [95% Conf. Interval]

-------------+----------------------------------------------------------------

time |

2 | 1 (empty)

3 | 1.332723 1.24129 0.31 0.758 0.2147511 8.270733

4 | 1 (empty)

5 | 1.432785 1.269832 0.41 0.685 0.252225 8.139054

|

BMI | 1.015713 0.0452353 0.35 0.726 0.9308123 1.108357

|

age binary |

> =40 years | **3.867011 4.322484 1.21 0.226 0.4324297 34.58081**

|

CD4cat |

< 200 | **0.3924614 0.421687 -0.87 0.384 0.0477759 3.223928**

_cons | 0.0452665 0.085947 -1.63 0.103 0.0010955 1.870445

------------------------------------------------------------------------------

Note: _cons estimates baseline risk.

## References

[1] UNAIDS The gap report [homepage on the Internet]. Geneva, Switzerland: Joint United Nations Programme on HIV/AIDS (UNAIDS); 2014 17 November 2015. Report No.: Contract No.: 18 November. [cited 2018 Jan 11]. Available from: http://files.unaids.org/en/media/unaids/contentassets/documents/unaidspublication/2014/UNAIDS_Gap_report_en.pdf

[2] Nel A, Mabude Z, Smit J, et al HIV incidence remains high in KwaZulu-Natal, South Africa: Evidence from three districts. PLoS One. 2012;7(4):1–8. https://doi.org/10.1371/journal.pone.0035278 10.1371/journal.pone.0035278 PMC3334938 22536364

[3] HIV/AIDS JUNPo, HIV/AIDS JUNPo 90-90-90: An ambitious treatment target to help end the AIDS epidemic [homepage on the Internet]. Geneva: UNAIDS; 2014 [cited 2018 Jan 11]. Available from: http://www.unaids.org/sites/default/files/media_asset/90-90-90_en_0.pdf

[4] Bhimma R, Adhikari M, Asharam K, Connolly C The spectrum of chronic kidney disease (stages 2–5) in KwaZulu-Natal, South Africa. Pediatr Nephrol. 2008;23(10):1841–1846. https://doi.org/10.1007/s00467-008-0871-5 18548285 10.1007/s00467-008-0871-5

[5] Young F, Critchley JA, Johnstone LK, Unwin NC A review of co-morbidity between infectious and chronic disease in Sub Saharan Africa: TB and diabetes mellitus, HIV and metabolic syndrome, and the impact of globalization. Global Health. 2009;5(1):9 https://doi.org/10.1186/1744-8603-5-9 19751503 10.1186/1744-8603-5-9 PMC2753337

[6] Stanifer JW, Jing B, Tolan S, et al The epidemiology of chronic kidney disease in sub-Saharan Africa: A systematic review and meta-analysis. Lancet Global Health. 2014;2(3):e174–e181. https://doi.org/10.1016/S2214-109X(14)70002-6 25102850 10.1016/S2214-109X(14)70002-6

[7] Kalyesubula R, Perazella MA Nephrotoxicity of HAART. AIDS Res Treat. 2011;2011:1–11. https://doi.org/10.1155/2011/562790 10.1155/2011/562790 PMC3157198 21860787

[8] Izzedine H, Harris M, Perazella MA The nephrotoxic effects of HAART. Nat Rev Nephrol. 2009;5(10):563–573. https://doi.org/10.1038/nrneph.2009.142 19776778 10.1038/nrneph.2009.142

[9] Daugas E, Rougier J-P, Hill G HAART-related nephropathies in HIV-infected patients. Kidney Int. 2005;67(2):393–403. https://doi.org/10.1111/j.1523-1755.2005.67096.x 15673287 10.1111/j.1523-1755.2005.67096.x

[10] Jafari A, Khalili H, Dashti-Khavidaki S Tenofovir-induced nephrotoxicity: Incidence, mechanism, risk factors, prognosis and proposed agents for prevention. Eur J Clin Pharmacol. 2014;70(9):1029–1040. https://doi.org/10.1007/s00228-014-1712-z 24958564 10.1007/s00228-014-1712-z

[11] Herlitz LC, Mohan S, Stokes MB, Radhakrishnan J, D’Agati VD, Markowitz GS Tenofovir nephrotoxicity: Acute tubular necrosis with distinctive clinical, pathological, and mitochondrial abnormalities. Kidney Int. 2010;78(11):1171–1177. https://doi.org/10.1038/ki.2010.318 20811330 10.1038/ki.2010.318

[12] Brennan A, Evans D, Maskew M, et al Relationship between renal dysfunction, nephrotoxicity and death among HIV adults on tenofovir. AIDS (London, England). 2011;25(13):1603 https://doi.org/10.1097/QAD.0b013e32834957da 10.1097/QAD.0b013e32834957da PMC3586413 21646902

[13] National Department of Health South African antiretroviral treatment guidelines 2010 [homepage on the Internet]. 2010 [cited 2018 Jan 11]. Available from: http://apps.who.int/medicinedocs/documents/s19153en/s19153en.pdf

[14] Levey AS, Eckardt K-U, Tsukamoto Y, et al Definition and classification of chronic kidney disease: A position statement from Kidney Disease: Improving Global Outcomes (KDIGO). Kidney Int. 2005;67(6):2089–2398. https://doi.org/10.1111/j.1523-1755.2005.00365.x 15882252 10.1111/j.1523-1755.2005.00365.x

[15] Van Deventer HE, George JA, Paiker JE, Becker PJ, Katz IJ Estimating glomerular filtration rate in black South Africans by use of the modification of diet in renal disease and Cockcroft-Gault equations. Clin Chem. 2008;54(7):1197–1202. https://doi.org/10.1373/clinchem.2007.099085 18487286 10.1373/clinchem.2007.099085

[16] Seedat YK, Rayner BL, Veriava Y South African hypertension practice guideline 2014. Cardiovasc J Afr. 2014;25(6):288–294. https://doi.org/10.5830/CVJA-2014-062023 25629715 10.5830/CVJA-2014-062 PMC4327181

[17] Bell K, Twiggs J, Olin BR, Date IR Hypertension: The silent killer: Updated JNC-8 guideline recommendations [homepage on the Internet]. Montgomery, AL: Alabama Pharmacy Association; 2015 [cited 2018 Jan 11]. Available from: http://c.ymcdn.com/sites/www.aparx.org/resource/resmgr/CEs/CE_Hypertension_The_Silent_K.pdf

[18] World Health Organization Global Database on Body Mass Index [homepage on the Internet]. [cited 2018 Jan 11]. Available from: http://apps.who.int/bmi/index.jsp?introPage=intro_3.html

[19] Kamkuemah M, Kaplan R, Bekker LG, Little F, Myer L Renal impairment in HIV-infected patients initiating tenofovir-containing antiretroviral therapy regimens in a primary healthcare setting in South Africa. Trop Med Int Health. 2015;20(4):518–526. https://doi.org/10.1111/tmi.12446 25442109 10.1111/tmi.12446

[20] Franey C, Knott D, Barnighausen T, et al Renal impairment in a rural African antiretroviral programme. BMC Infect Dis. 2009;9(1):143 https://doi.org/10.1186/1471-2334-9-143 19715590 10.1186/1471-2334-9-143 PMC2743696

[21] Fabian J, Naicker S, Goetsch S, Venter WD The clinical and histological response of HIV-associated kidney disease to antiretroviral therapy in South Africans. Nephrol Dial Transplant. 2013;28(6):1543–1554. https://doi.org/10.1093/ndt/gft010 23444185 10.1093/ndt/gft010

[22] Wearne N, Swanepoel CR, Boulle A, Duffield MS, Rayner BL The spectrum of renal histologies seen in HIV with outcomes, prognostic indicators and clinical correlations. Nephrol Dial Transplant. 2012;27(11):4109–4118. https://doi.org/10.1093/ndt/gfr702 22200584 10.1093/ndt/gfr702

[23] Stohr W, Reid A, Walker AS, et al Glomerular dysfunction and associated risk factors over 4–5 years following antiretroviral therapy initiation in Africa. Antivir Ther. 2011;16(7):1011–1020. https://doi.org/10.3851/IMP1832 22024517 10.3851/IMP1832

[24] Krawczyk CS, Holmberg SD, Moorman AC, et al Factors associated with chronic renal failure in HIV-infected ambulatory patients. AIDS. 2004;18(16):2171–2178. https://doi.org/10.1097/00002030-200411050-00009 15577650 10.1097/00002030-200411050-00009

[25] Ross MJ, Klotman PE Recent progress in HIV-associated nephropathy. J Am Soc Nephrol. 2002;13(12):2997–3004. https://doi.org/10.1097/01.ASN.0000040750.40907.99 12444220 10.1097/01.asn.0000040750.40907.99

[26] Déti EK, Thiébaut R, Bonnet F, et al Prevalence and factors associated with renal impairment in HIV-infected patients, ANRS C03 Aquitaine Cohort, France. HIV Med. 2010;11(5):308–317. https://doi.org/10.1111/j.1468-1293.2009.00780.x 20002500 10.1111/j.1468-1293.2009.00780.x

[27] Reid A, Stöhr W, Walker AS, et al Severe renal dysfunction and risk factors associated with renal impairment in HIV-infected adults in Africa initiating antiretroviral therapy. Clin Infect Dis. 2008;46(8):1271–1281. https://doi.org/10.1086/533468 18444867 10.1086/533468

[28] Field AE, Coakley EH, Must A, et al Impact of overweight on the risk of developing common chronic diseases during a 10-year period. Arch Intern Med. 2001;161(13):1581–1586. https://doi.org/10.1001/archinte.161.13.1581 11434789 10.1001/archinte.161.13.1581

[29] Chobanian AV, Bakris GL, Black HR, et al The seventh report of the joint national committee on prevention, detection, evaluation, and treatment of high blood pressure: The JNC 7 report. JAMA. 2003;289(19):2560–2571. https://doi.org/10.1001/jama.289.19.2560 12748199 10.1001/jama.289.19.2560

[30] Cooper RD, Wiebe N, Smith N, Keiser P, Naicker S, Tonelli M Systematic review and meta-analysis: Renal safety of tenofovir disoproxil fumarate in HIV-infected patients. Clin Infect Dis. 2010;51(5):496–505. https://doi.org/10.1086/655681 20673002 10.1086/655681

[31] Elias A, Ijeoma O, Edikpo NJ, Oputiri D, Geoffrey O-BP Tenofovir renal toxicity: Evaluation of cohorts and clinical studies – Part 2. Pharmacol Pharm. 2014;5(1):97–111. https://doi.org/10.4236/pp.2014.51015

[32] National Department of Health South African antiretroviral treatment guidelines [homepage on the Internet]. National Department of Health (South Africa); 2015 [cited 2018 Jan 11]. Available from: http://www.sahivsoc.org/Files/ART%20Guidelines%2015052015.pdf

[33] Fabian J, Naicker S, Venter WD, et al Urinary screening abnormalities in antiretroviral-naive HIV-infected outpatients and implications for management – A single-center study in South Africa. Ethn Dis. 2009;19(1):80. 19484882

[34] Siedner MJ, Atta MG, Lucas GM, Perazella MA, Fine DM Poor validity of urine dipstick as a screening tool for proteinuria in HIV-positive patients. J Acquir Immune Defic Syndr. 2008;47(2):261–263. https://doi.org/10.1097/QAI.0b013e31815ac4ad 18223364 10.1097/QAI.0b013e31815ac4ad

[35] Han T, Naicker S, Ramdial P, Assounga A A cross-sectional study of HIV-seropositive patients with varying degrees of proteinuria in South Africa. Kidney Int. 2006;69(12):22432250 https://doi.org/10.1038/sj.ki.5000339 10.1038/sj.ki.5000339 16672914

[36] D’Agati V, Appel GB HIV infection and the kidney. J Am Soc Nephrol. 1997;8(1):138–152. 9013459 10.1681/ASN.V81138

[37] Kasembeli AN, Duarte R, Ramsay M, et al APOL1 risk variants are strongly associated with HIV-associated nephropathy in black South Africans. J Am Soc Nephrol. 2015;26(11):2882–2890. https://doi.org/10.1681/ASN.2014050469 25788523 10.1681/ASN.2014050469 PMC4625661

[38] Sax PE, Wohl D, Yin MT, et al Tenofovir alafenamide versus tenofovir disoproxil fumarate, coformulated with elvitegravir, cobicistat, and emtricitabine, for initial treatment of HIV-1 infection: Two randomised, double-blind, phase 3, non-inferiority trials. Lancet. 2015;385(9987):2606–2615. https://doi.org/10.1016/S0140-6736(15)60616-X 25890673 10.1016/S0140-6736(15)60616-X

